# The use of thermal imaging for monitoring the training progress of professional male sweep rowers

**DOI:** 10.1038/s41598-022-20848-7

**Published:** 2022-10-03

**Authors:** Anna Straburzyńska-Lupa, Paweł Korman, Ewa Śliwicka, Jakub Kryściak, Małgorzata Barbara Ogurkowska

**Affiliations:** 1 Department of Physical Therapy and Sports Recovery, Poznan University of Physical Education, Królowej Jadwigi Str. 27/39, 61-871 Poznań, Poland; 2 Department of Physiology and Biochemistry, Poznan University of Physical Education, Poznań, Poland; 3 Department of Biomechanics, Poznan University of Physical Education, Poznań, Poland

**Keywords:** Biophysics, Physiology

## Abstract

This study assesses the thermal profile of the skin in highly trained rowers and investigates the relationship between resting skin temperature (Ts) and the muscle peak torque (PT) measured in statics at the beginning (autumn) and the end (spring) of the preparatory period. Ten professional male sweep rowers, members of the Polish national rowing team, were investigated. A thermal imaging camera was used to analyze the Ts. The PT of the muscles involved in the rowing cycle were measured isometrically. No significant temperature asymmetries were found, except in front of arms after exercise in the spring (*p* = 0.0228). In contrast, the PT test in the autumn confirmed the significant asymmetry of the knee joint extensors (*p* = 0.0192). In spring compared to autumn, Ts in many areas of the body were slightly higher, as was PT of underlying muscles. Significant correlations between resting Ts and PT of the underlying muscles were found. Thermal imaging makes it possible to observe changes in skin temperature and symmetry before and after exercise. At this stage, it does not appear to be a method that, without supporting of other methods such as those assessing muscle function, will allow monitoring of training progress.

## Introduction

In professional sport great attention is paid to monitoring training progress in terms of optimally balancing training loads and subsequent recovery to achieve high performance and minimize the risk of extreme fatigue, overtraining, and adverse health effects, like injury, illness, and burnout^1^. It becomes crucial to define the fine line between a high and excessive training loads, and therefore it is important to monitor the training loads and recovery^1^. For this purpose many methods, including physiological, biochemical, biomechanical, or psychological are used^1–6^.

Elite rowing is a sport requiring intensive training which, in addition to sessions on the water, involves strength and power endurance, endurance workout, gymnastics and exercises improving velocity and agility^1,2^. Rowing involves the majority of muscle groups in the body (over 70%)^7^, and during exercise muscular metabolism increases by 5 to 15 times to provide energy for skeletal muscle contraction, when compared to the resting rate. Because 70 to 100 percent of the metabolism is released as heat, therefore it requires effective homeostatic thermoregulatory mechanisms that prevent hyperthermia and release excess heat from the body^8^. The preparatory period is particularly demanding due to the large numbers and intensity of training sessions^7^. The intense nature of rowing training makes them particularly predisposed to imbalances between the applied training loads and recovery, hence the need to look for appropriate monitoring tools^1,2^.

Physical exertion requires efficient thermoregulation mechanisms to minimize changes in core temperature and to maintain physiological homeostasis^9^. The greatest changes take place in the skin, muscle and core tissue^9^. During intense and continuous physical exercise, the core temperature rises due to increased metabolic heat production^10^. Blood circulation of the skin is temporarily reduced as blood is directed primarily to the working muscles^10–12^ to ensure adequate temperature required for optimal strength and endurance performance of working muscles^10^, and skin temperature usually decreases^12^. When the core temperature reaches the specific target value^10^, heat loss mechanisms are activated^10,13^ such as vasodilation, increased cutaneous blood flow, and sweating, to prevent tissue temperature from rising excessively^9^. It is assumed that blood flow in the skin, regulated by the sympathetic neural control, reflects the thermoregulation processes inside the body^10,14^. These mechanisms are designed to dissipate extra heat production from the core and contracting muscles^15^, so that skin temperatures may rise^16^. It is considered that the thermal balance between muscle engagement, activity of circulatory system recruitment level and sweat evaporation rate determines the skin temperature in each region of the body^16^.

An important indicator of the body's heat load during exercise is core temperature^17^, which shows a direct relationship with exercise intensity^16,18^. For research purposes, ingestible telemetry pills are now increasingly used, as an alternative to measuring esophageal and rectal temperature^19^. However, monitoring core temperature in active individuals has some limitations^16^, and its values vary among internal measurement sites^18^. Hence the interest in measurement of skin temperature has been expressed by many researchers^10,16,20,21^, especially since it allows monitoring of local temperature changes linked to, among other things, the activity of superficially lying muscles. At the same time, the study by Priego Quesada et al.^16^ showed various correlations between the temperature of different skin regions and core.

It should be noted that muscle strength and power are important elements in the physical preparation of athletes. A significant relationship is found between maximal power (or force) and sports performance^5,22^. The intramuscular temperature is an important factor influencing the ability of muscles to generate maximal power^23^. Its magnitude at rest, measured at a depth of about 4 cm in the quadriceps muscle of the thigh, is about 36 °C, which leads to an increase in the ability to generate a maximum power of about 15–20%. As a result of the temperature increase, the maximum shortening rate of muscle fibers increases. Furthermore, Bergh and Ekblom^24^ reported that isometric force changes by 2% per 1 °C. Thus, this effect is rather small, although important in sport, because if the muscle temperature increases by 3–4 °C after a proper warm-up, the isometric force increases by about 8%, and the maximum power generated by the muscles increases by about 15–20%.

Infrared thermography (IRT) allows visualization of temperature distribution on the surface of the human body and monitoring changes that occur in response to physiological or pathological processes in the body^25,26^. Research on the possibility of use of IRT as a convenient and non-invasive method^20,27^ in sport has been ongoing for several years^25–31^. Skin temperature has been shown to be related to exercise intensity^32^ and physical fitness^33^. Studies demonstrated that endurance training induces adaptation changes in the skin blood flow response to exercise^13^. Changes in skin temperature using IRT associated with muscle and joint activity and recovery have been observed^21^. However, the relationship between muscular activity and skin temperature still requires further research^16^.^.^ Therefore, the purpose of this study was to evaluate the thermal profile of the skin in highly trained rowers and to investigate the relationship between resting skin temperature (Ts) and the muscle peak torque (PT) measured in statics at the beginning and the end of the preparatory period in connection with an attempt to demonstrate the applicability of thermal imaging measurements to assess the stabilization capacity of muscles working during rowing.

The research was conducted as part of a major project on evaluating the impact of changes in musculoskeletal overload on athletic performance during the annual training cycle in asymmetric athletes in the context of training optimization.

## Results

The main characteristics of the rowers are summarized in Table 1. No significant differences were observed for maximal oxygen consumption (VO_2_max), body mass, fat mass, fat, and free fat mass between examination at the beginning and at the end of the preparatory period.

**Table 1:** Characteristics of the group (rowers *n* = 10).

Variables	I examination	II examination	*p*—value
Age [years]	20.6 ± 1.3; 20.5 (19.0 ÷ 21.0)		
Training experience [years]	7.3 ± 1.6; 7.0 (6.0 ÷ 8.0)		
Body height [cm]	191 ± 0.04; 190 (189 ÷ 193)		
Body mass [kg]	91.8 ± 7.41; 91.7 (89.5 ÷ 95.5)	91.6 ± 7.64; 92.4 (88.2 ÷ 94.0)	0.8261
Fat mass [kg]	11.4 ± 3.24; 11.7 (9.3 ÷ 13.3)	10.8 ± 3.76; 10.4 (8.3 ÷ 12.6)	0.3875
Fat [%]	12.3 ± 2.74; 13.1 (9.9 ÷ 13.9)	11.6 ± 3.27; 11.6 (8.8 ÷ 13.3)	0.3965
FFM [kg]	80.4 ± 5.08; (77.4 ÷ 82.9)	80.9 ± 5.76; 81.6 (79.5 ÷ 85.7)	0.4148
VO_2_max [ml ∙ kg^-1^ ∙ min^-1^]	59.6 ± 2.94; 57.2 (56.9 ÷ 59.9)	58.7 ± 3.60; 58.2 (57.0 ÷ 60.6)	0.1842

Data are presented as mean ± SD, Median (Q_1_ ÷ Q_3_). FFM, fat free mass; VO_2_max, maximal oxygen uptake.

### Symmetry of skin temperature at rest and after graded exercise testing in the autumn (I) and spring (II) examination

No significant temperature asymmetries were found in the examinations in two terms during graded exercise testing (pre-, post-exercise and in recovery), except front part of arms immediately after exercise in spring (*p* = 0.0228).

The difference of temperature measured at rest in autumn (I) did not exceed 0.15 °C for the front part of upper and lower extremities, 0.17 °C for the back part; 0.04 °C for the front part of the trunk, 0.1 °C for the back part; and in spring (II) 0.07 °C for the front of upper and lower extremities, 0.42 °C for the back; 0.07 °C for the front of the trunk, 0.09 °C for the back.

The difference of temperature measured in autumn (I) immediately post-exercise did not exceed 0.08 °C for the front part of upper and lower extremities, 0.7 °C for the back part; 0.32 °C for the front part of the trunk, 0.19 °C for the back part; and in spring (II) 0.22 °C for the front part of upper and lower extremities, 0.21 °C for the back part; 0.17 °C for the front part of the trunk, 0.07 °C for the back part.

The difference of recovery temperature measured in autumn (I) in recovery did not exceed 0.37 °C for the front part of upper and lower extremities, 0.61 °C for the back part; 0.14 °C for the front part of the trunk, 0.06 °C for the back part, and in spring (II) 0.25 °C for the front part of upper and lower extremities, 0.57 °C for the back part; 0.15 °C for the front part of the trunk, 0.1 °C for the back part.

### Skin temperature time course after graded exercise testing and in recovery in the autumn (I) and spring (II) examinations in the front of the body

**Figure 1:**
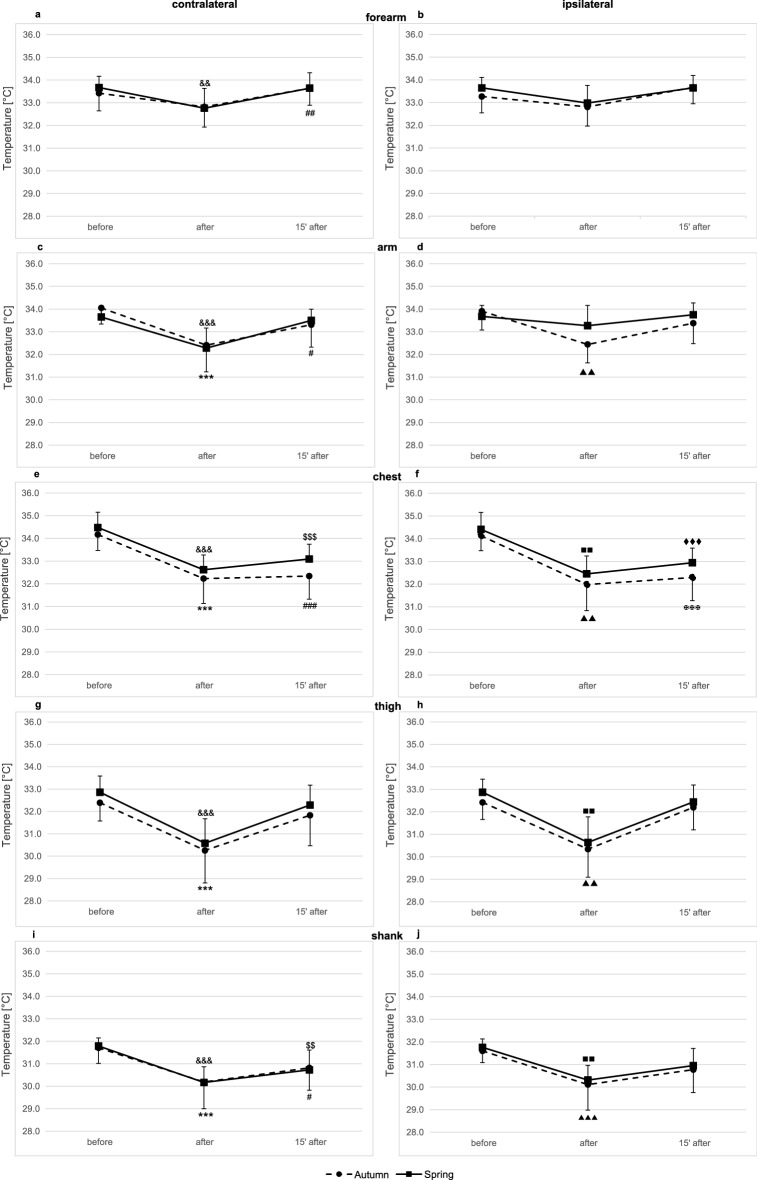
Skin surface temperature of the front side of the body. (**a**) contralateral forearm, (**b**) ipsilateral forearm, (**c**) contralateral arm, (**d**) ipsilateral arm, (**e**) contralateral side of the chest, (**f**) ipsilateral side of the chest, (**g**) contralateral thigh, (**h**) ipsilateral thigh, (**i**) contralateral shank, (**j**) ipsilateral shank. Data are presented as means. ^***^p ≤ 0.001 significant differences between measurements before and after exercise test in autumn on the contralateral side of the body. ^###^*p* ≤ 0.001 significant differences between measurements before and 15 min after exercise test in autumn on the contralateral side of the body. ^##^*p* ≤ 0.01 significant differences between measurements before and 15 min after exercise test in autumn on the contralateral side of the body. ^#^*p* < 0.5 significant differences between measurements before and 15 min after exercise test in autumn on the contralateral side of the body. ^&&&^*p* ≤ 0.001 significant differences between measurements before and after exercise test in spring on the contralateral side of the body. ^&&^*p* ≤ 0.01 significant differences between measurements before and after exercise test in spring on the contralateral side of the body. ^$$$^*p* ≤ 0.001 significant differences between measurements before and 15 min after exercise test in spring on the contralateral side of the body. ^$$^*p* ≤ 0.01 significant differences between measurements before and 15 min after exercise test in spring on the contralateral side of the body. ^▲▲▲^*p* ≤ 0.001 significant differences between measurements before and after exercise test in autumn on the ipsilateral side. ^✠✠✠^*p* ≤ 0.001 significant differences between measurements before and 15 min after exercise test in autumn on the ipsilateral side of the body. ^■■■^*p* ≤ 0.01 significant differences between measurements before and after exercise test in Spring on the ipsilateral side of the body. ^♦♦♦^*p* ≤ 0.01 significant differences between measurements before and 15 min after exercise test in spring on the ipsilateral side of the body.

**Figure 2:**
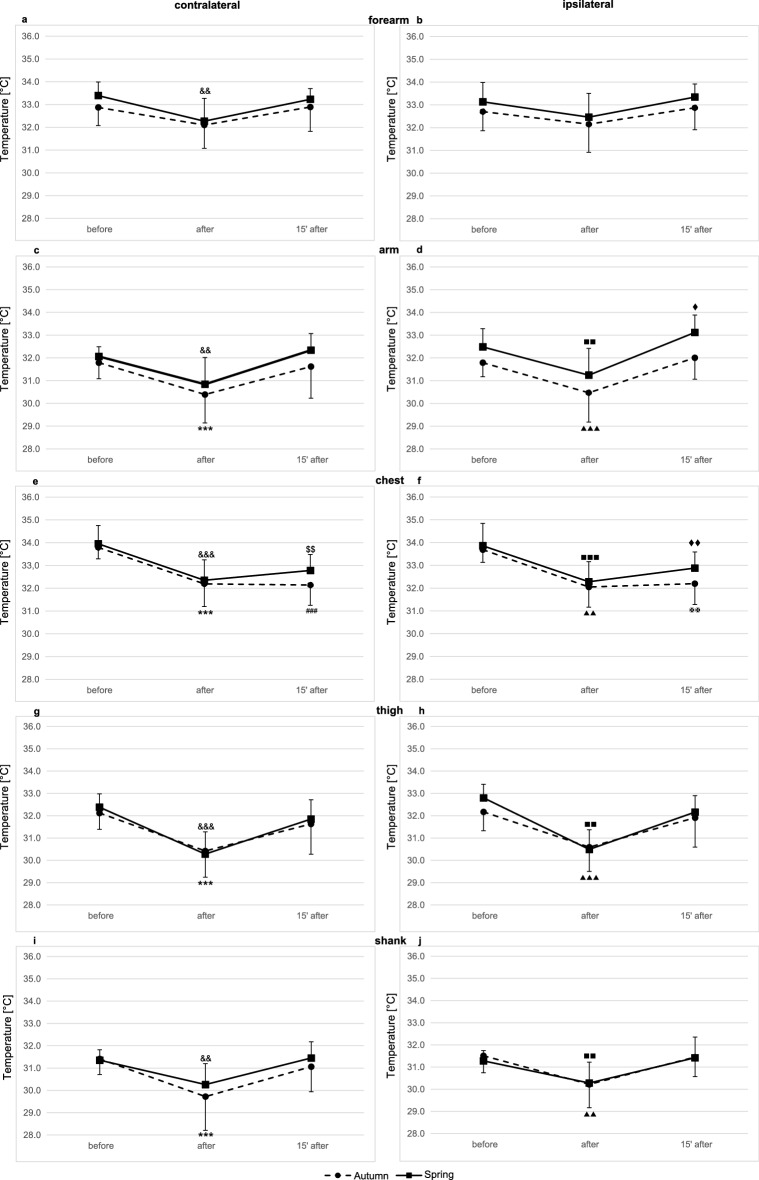
Skin surface temperature of the back side of the body. (**a**) contralateral forearm, (**b**) ipsilateral forearm, (**c**) contralateral arm, (**d**) ipsilateral arm, (**e**) contralateral side of the chest, (**f**) ipsilateral side of the chest, (**g**) contralateral thigh, (**h**) ipsilateral thigh, (**i**) contralateral shank, (**j**) ipsilateral shank. Data are presented as means. ^***^p ≤ 0.001 significant differences between measurements before and after exercise test in autumn on the contralateral side. ^###^*p* ≤ 0.001 significant differences between measurements before and 15 min after exercise test in autumn on the contralateral side of the body. ^&&&^*p* ≤ 0.001 significant differences between measurements before and after exercise test in spring on the contralateral side of the body. ^&&^*p* ≤ 0.01 significant differences between measurements before and after exercise test in spring on the contralateral side of the body. ^$$^*p* ≤ 0.01 significant differences between measurements before and 15 min after exercise test in spring on the contralateral side of the body. ^▲▲▲^*p* ≤ 0.001 significant differences between measurements before and after exercise test in autumn on the ipsilateral side. ^✠✠✠^*p* ≤ 0.001 significant differences between measurements before and 15 min after exercise test in autumn on the ipsilateral side of the body. ^■■■^*p* ≤ 0.001 significant differences between measurements before and after exercise test in spring on the ipsilateral side of the body. ^■■^*p* ≤ 0.01 significant differences between measurements before and after exercise test in spring on the ipsilateral side of the body. ^♦♦^*p* ≤ 0.01 significant differences between measurements before and 15 min after exercise test in spring on the ipsilateral side of the body. ^♦^*p* ≤ 0.05 significant differences between measurements before and 15 min after exercise test in spring on the ipsilateral side of the body.

The effects of the graded exercise test on skin temperature in the front of the body in the autumn and in the spring are presented in Figs. 1 and 2.

In the front of the body significant time-effects were noted in the following locations: forearm (contralateral: *p* = 0.0000; ipsilateral: *p* = 0.0001), arm (contralateral: *p* = 0.0000; ipsilateral: *p* = 0.0000), chest (contralateral: p = 0.0000; ipsilateral: *p* = 0.0000), thigh (contralateral: *p* = 0.0000; ipsilateral: *p* = 0.0000), shank (contralateral: *p* = 0.0000; ipsilateral: *p* = 0.0000). In the case of ipsilateral arm, repeated measures ANOVA revealed Examination × Time interaction, which suggested that there was a differential effect of the graded exercise test on skin temperature and recovery between the terms of examinations (Table 2).

**Table 2:** Summary of ANOVA results on effects of graded exercise test on skin temperature in the front of the body in autumn (I examination) and spring (II examination).

	Front			Back		
	F	*p*	*ƞ*^2^	F	*p*	*ƞ*^2^
**Contralateral forearm**						
Examination	0.05	0.8315	0.0026	1.12	0.3045	0.0584
Time	20.29	0.0000	0.5299	17.89	0.0000	0.4985
Examination x Time	0.63	0.5373	0.0339	0.49	0.6141	0.0267
**Ipsilateral forearm**						
Examination	0.49	0.4945	0.0263	1.24	0.2794	0.0646
Time	13.32	0.0000	0.4252	10.21	0.0003	0.3620
Examination x Time	0.85	0.4372	0.0449	0.10	0.9039	0.0056
**Contralateral arm**						
Examination	0.12	0.7287	0.0068	1.59	0.2233	0.0812
Time	46.46	0.0000	0.7208	27.23	0.0000	0.6021
Examination x Time	1.70	0.1979	0.0861	0.58	0.5623	0.0315
**Ipsilateral arm**						
Examination	1.38	0.2556	0.0711	2.46	0.1343	0.1202
Time	16.07	0.0000	0.4717	49.79	0.0000	0.7345
Examination x Time	4.73	0.0150	0.2080	1.55	0.2253	0.0795
**Contralateral side of the chest**				**Contralateral side of the back**		
Examination	0.38	0.5470	0.0205	1.18	0.2918	0.0615
Time	58.34	0.0000	0.7642	44.47	0.0000	0.7119
Examination x Time	0.78	0.4656	0.0416	1.12	0.3882	0.0585
**Ipsilateral side of the chest**				**Ipsilateral side of the back**		
Examination	0.47	0.5033	0.0253	1.37	0.2571	0.0707
Time	66.90	0.0000	0.7880	42.19	0.0000	0.7010
Examination x Time	0.72	0.4933	0.0385	1.15	0.3265	0.0603
**Contralateral thigh**						
Examination	0.94	0.3454	0.0496	0.08	0.7745	0.0047
Time	26.24	0.0000	0.7821	59.11	0.0000	0.7666
Examination x Time	0.09	0.9172	0.0048	0.74	0.4835	0.0396
**Ipsilateral thigh**						
Examination	0.87	0.3642	0.0459	0.56	0.4622	0.0304
Time	69.09	0.0000	0.7933	48.11	0.0000	0.7277
Examination x Time	0.15	0.8615	0.0082	1.55	0.2268	0.0791
**Contralateral shank**						
Examination	0.00	0.9742	0.0001	0.59	0.4523	0.0317
Time	36.71	0.0000	0.6710	33.83	0.0000	0.6527
Examination x Time	0.11	0.8991	0.0059	1.39	0.2610	0.0719
**Ipsilateral shank**						
Examination	0.42	0.5256	0.0227	0.04	0.8424	0.0023
Time	30.88	0.0000	0.6318	30.52	0.0000	0.6291
Examination x Time	0.01	0.9943	0.0003	0.37	0.6924	0.0202

In both examinations a significant drop in temperature compared to the baseline (before vs after) was observed immediately after exercise in all areas measured, except contralateral forearm (autumn: *p* = 0.0946), ipsilateral forearm (autumn: *p* = 0.6205; spring: *p* = 0.0590), and ipsilateral arm (spring: *p* = 1.0000).

In recovery (15 min after exercise) the temperature returned to baseline levels (before vs. 15′ after). A significant change in temperature was observed in relation to baseline values at the following locations: ipsilateral arm (autumn: *p* = 0.0364), contralateral side of the chest (autumn: *p* = 0.0000; spring: *p* = 0.0001), ipsilateral side of the chest (autumn: *p* = 0.0000; spring: *p* = 0.0001) and contralateral shank (autumn: *p* = 0.0248; spring: *p* = 0.0039).

### Skin temperature time course after graded exercise testing and in recovery in autumn (I) and spring (II) examinations in the back of the body

The effects of the graded exercise test on skin temperature in the back of the body in the autumn and in the spring are presented in Figs. 1 and 2.

In the back of the body significant time-effects were noted in the following locations: forearm (contralateral: *p* = 0.0000; ipsilateral: *p* = 0.0003), arm (contralateral: *p* = 0.0000; ipsilateral: *p* = 0.0000), back (contralateral: *p* = 0.0000; ipsilateral: *p* = 0.0000), thigh (contralateral: *p* = 0.0000; ipsilateral: *p* = 0.0000), shank (contralateral: *p* = 0.0.0000; ipsilateral: *p* = 0.0000) (Table 2).

In both examinations a significant drop in temperature compared to the baseline (before vs. after) was observed immediately after exercise in all areas measured, except contralateral forearm (autumn: *p* = 0.0573) and ipsilateral forearm (autumn: *p* = 0.6383; spring: *p* = 0.2214).

In recovery (15 min after exercise) the temperature returned to baseline levels (before vs. 15′ after). A significant change in temperature was observed in relation to baseline values at the following locations: ipsilateral arm (spring: *p* = 0.0451), contralateral side of the back (autumn: *p* = 0.0000; spring: *p* = 0.0011) and ipsilateral side of the back (autumn: *p* = 0.0000; spring: *p* = 0.0086).

### Comparison of skin temperature baseline and after graded exercise testing and in recovery in autumn (I) to spring (II) examinations (front and back)

No significant differences in skin temperature were observed in resting, post-exercise and recovery in spring compared to autumn.

### Comparison of muscle forces and peak torques measured in statics at the beginning (I) and the end (II) of the preparatory period

Comparison of muscle forces and peak torques at the beginning (autumn) and the end (spring) of the preparatory period are shown in Table 3. There were significant changes in peak torques developed by the flexor muscle at the ipsilateral shoulder joint (*p* = 0.0219) and increases in PT of the trunk extensors (*p* = 0.0134). In contrast, a tendency for increases in PT between the beginning and the end of the preparatory period for the ipsilateral hip flexor (*p* = 0.0932), contralateral flexor muscles (*p* = 0.0738), and ipsilateral (*p* = 0.0934) of the knee joint, as well as the contralateral rotator of the trunk (*p* = 0.0738) were observed. At the same time, a tendency for a decrease in relative lower limb ipsilateral global strength was also observed (p = 0.0927).

**Table 3:** Comparison of muscle forces and peak torques at first and second examination.

Variables	I examination	II examination	*p*—value
GSLL-CE [N/kg]	27.58 ± 4.75; 28.08 (26.34 ÷ 29.19)	22.89 ± 5.64; 24.03 (17.68 ÷ 26.82)	0.0927
HJT-IF [Nm/kg]	1.63 ± 0.46; 1.52 (1.33, 1.99)	1.89 ± 0.42; 1.92 (1.67 ÷ 2.11)	0.0932
KJT-CF [Nm/kg]	1.95 ± 0.40; 1.84 (1.64, 2.32)	2.14 ± 0.39; 2.12 (1.80 ÷ 2.37)	0.0738
KJT-IF [Nm/kg]	1.97 ± 0.50; 1.86 (1.61, 2.41)	2.19 ± 0.41; 2.23 (1.76 ÷ 2.36)	0.0934
SJT-IF [Nm/kg]	1.24 ± 0.21; 1.27 (1.08, 1.35)	1.03 ± 0.18; 0.96 (0.93 ÷ 1.22)	0.0219
TT-F [Nm/kg]	6.47 ± 0.90; 6.43 (5.83, 6.58)	7.10 ± 0.85; 7.07 (6.42 ÷ 7.83)	0.0134
TT-CR [Nm/kg]	1.01 ± 0.20; 1.02 (0.85, 1.13)	1.16 ± 0.27; 1.11 (1.03, ÷ 1.22)	0.0738

Data are presented as mean ± SD; median (Q_1_ ÷ Q_3_); GSLL-CE: global strength of lower limb—contralateral extensors, HJT-IF: hip joint torque—ipsilateral flexors, KJT—CF/KJT—IF: knee joint torque—contralateral/ipsilateral flexors, SJT-IF: shoulder joint torque—ipsilateral flexors, TT-E: trunk torque extensors, TT-LR: trunk torque—contralateral rotators.

### Asymmetry of muscle peak torques measured in statics between contralateral and ipsilateral sides of the body

Comparison of muscle torques between contralateral and ipsilateral sides of the body is shown in Table 4. A significant asymmetry of peak torque was obtained for the extensors of the knee joint in the first examination (I) (*p* = 0.0192); peak torque on the ipsilateral side is greater than that on the contralateral side. An analogous effect was obtained for the elbow joint extensors on the ipsilateral side (*p* = 0.0219). A significant difference was also observed on the second examination (II) for the contralateral and ipsilateral trunk rotators (*p* = 0.0122), an increase in PT was noted on the contralateral side relative to the ipsilateral side. A tendency was observed in the assessment of asymmetry in the second testing period for the knee extensors (*p* = 0.0737) and hip flexors (*p* = 0.0737).

**Table 4:** Comparison of muscle peak torques between contralateral and ipsilateral side of the body.

Variables	Body contralateral side	Body ipsilateral side	*p*—value
**I examination**			
Knee joint torque, extension [Nm/kg]	3.35 ± 0.63; 3.27 (2.97 ÷ 3.94)	3.65 ± 0.55; 3.74; (2.91 ÷ 3.89)	0.0192*
Elbow joint torque, extension [Nm/kg]	1.36 ± 0.28; 1.41 (1.22 ÷ 1.48)	1.45 ± 0.27; 1.48; (1.23 ÷ 1.59)	0.0219*
**II examination**			
Knee joint torque, extension [Nm/kg]	3.71 ± 0.68; 3.80 (3.08 ÷ 4.14)	3.88 ± 0.74; 3.93; (3.48 ÷ 4.55)	0.0737**
Hip joint torque, flexion [Nm/kg]	1.96 ± 0.49; 2.07; (1.40 ÷ 2.22)	1.89 ± 0.42; 1.92; (1.67 ÷ 2.11)	0.0737**
Trunk torque, left/right rotation [Nm/kg]	1.16 ± 0.27; 1.11; (1.03 ÷ 1.22)	0.96 ± 0.23; 1.05; (0.79 ÷ 1.14)	0.0122*

Data are presented as mean ± SD; median (Q_1_ ÷ Q_3_).

### Correlation between the peak torques (PT) of selected muscles and the resting skin temperature over these muscles

**Figure 3:**
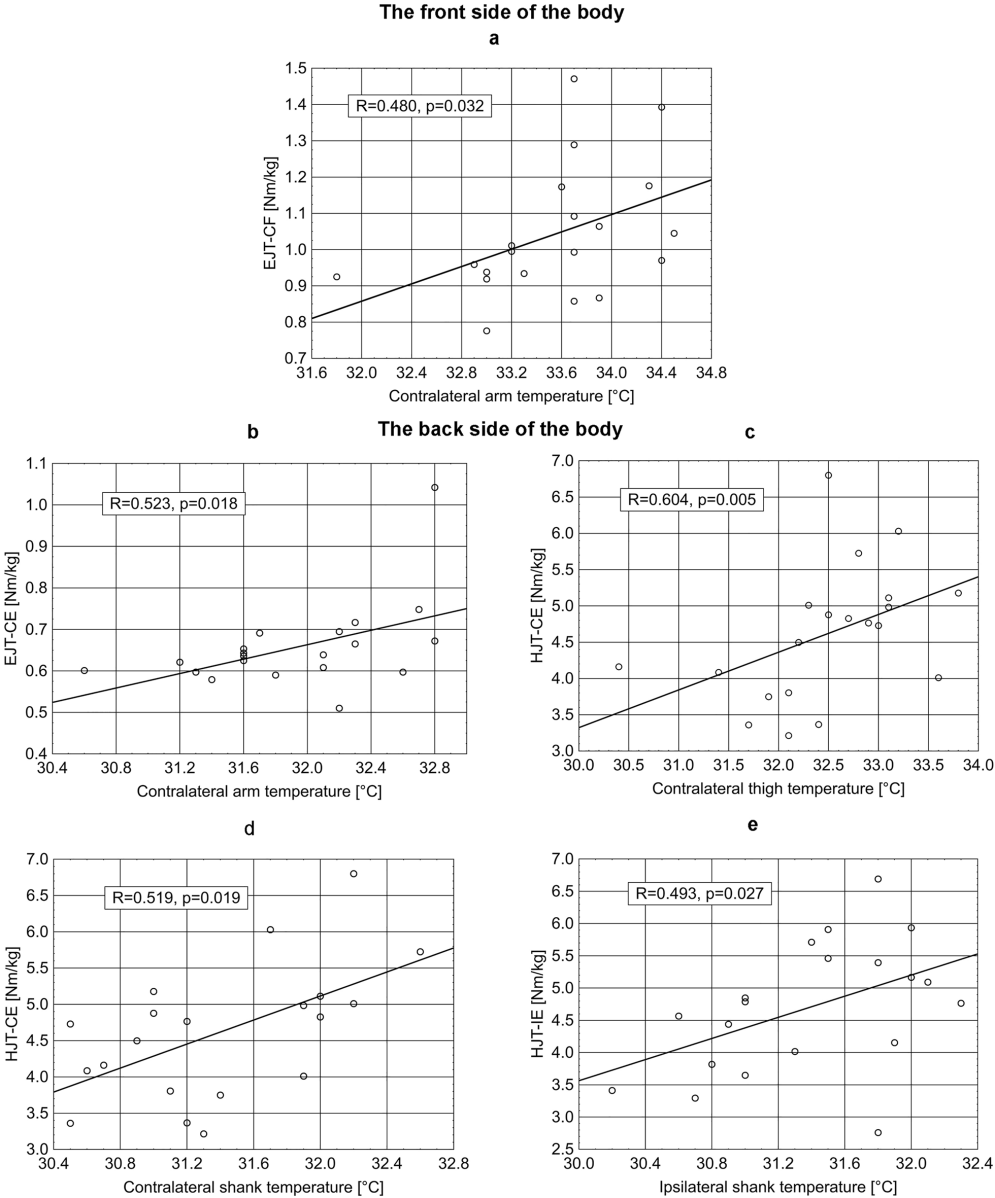
Correlations between the peak torques of selected muscles and the resting skin temperature measurement over these muscle. Spearman rank correlation test, *p* < 0:05 statistically significant value. EJT—CF: elbow joint torque—contralateral flexor. EJT—CE: elbow joint torque—contralateral extensor. HJT-CE: shank joint torque—contralateral extensor. HJT- IE: hip joint torque—ipsilateral extensor.

In the study group of competitive sweep rowers, there was a significant correlation between the peak torques (PT) of selected muscles and the resting skin temperature over these muscles. These relationships are shown in Fig. 3.

There is a significant correlation between the PT of the contralateral elbow flexor muscle and the temperature of the front of the contralateral arm (*p* = 0.0318), as well as between the PT of the contralateral elbow joint extensor muscle and the temperature of the back of the contralateral arm (*p* = 0.0184). A significant relationship was also established between the PT of the contralateral hip extensor muscle and the posterior temperature of the contralateral femur (*p* = 0.0049). The PT of the contralateral hip extensor muscle also correlates with the temperature of the back of the contralateral shank (*p* = 0.0187). A significant correlation exists between the PT of the ipsilateral hip extensor muscle and the temperature of the back of the ipsilateral shin (*p* = 0.0273).

## Discussion

To our best knowledge, our study is the first to evaluate the thermal profile of the skin in highly trained sweep rowers and to investigate the relationship between resting skin temperature (Ts) and the peak torque (PT) developed by muscles working during rowing at the beginning (autumn) and the end (spring) of the preparatory period. The major findings in this study were that no significant changes in Ts measured at symmetric sites at the beginning and the end of the preparatory period have been demonstrated, except the front part of arms immediately after exercise in spring. In contrast, the PT test performed at the beginning of the preparatory period confirmed the significant asymmetry of the knee joint extensors, which is linked to the biomechanics of rowing by sweep rowers. At the end of the preparatory period, compared to its beginning (autumn vs. spring), the surface temperature measured in many places was slightly higher (not significantly), as well as the PT over selected muscles. At the same time, we found significant correlations between the PT of selected muscles and the resting skin temperature measurement over these muscles.

So far, no thermal profile has been specified for any sport^27^. However, there are attempts to indicate pattern changes observed by infrared examination for each type of exercise, i.e. endurance exercise with a constant load, incremental exercise testing, and resistance exercise^34^. Taking into account the fact that skin blood flow can be modified by age, sex hormones, diseases, hydration^14^, and subcutaneous fat content^35^, our study concerned only young healthy, lean, and highly trained male sweep rowers during the preparatory period (Table 1).

In our study, it can be seen that higher temperature measured at rest is found in the front part of the body compared to the back. The highest temperatures were found in the chest and the lowest in the distal limbs, which is consistent with the results obtained by other authors^36^.

An important part of our study was also looking for a link between the resting Ts and the PT developed by muscles working during rowing. To our knowledge, to date, there are no human studies linking the measurement of PT to Ts. There are only studies that have used measurements of electrical muscle activity and combined them with skin temperature and internal rectal testing. These studies generally consist in cooling or heating the subject and looking for the effect of the temperature change on neuromuscular responses^37,38^. The relationship between neuromuscular activation and skin temperature during exercise, such as cycling, has also been assessed^16^.

In our study, we found significant correlations between the PT of selected muscles under isometric conditions, especially those that are shallow in relation to the skin surface on which the temperature was measured. For example, it was found that in the case of the flexor muscle of the elbow joint (*biceps brachii*), there is a significant relationship between its PT and the Ts at the anterior aspect of the arm. Moreover, the PT of the extensor muscle of this joint (*triceps brachii*) correlates with the temperature of the posterior side of the arm. Also in the case of the hip joint, a significant relationship was found between the temperature at the back of the thigh and the PT of the hip extensor. There is also a significant correlation of the temperature of the lower leg as a mobile member of the knee joint with the torque of the hip extensor. At the same time, it should be noted that the above relationships are only possible when considering muscles located shallowly in relation to the skin surface. Such relationships do not exist for deep muscles, such as the spinal column. It should be emphasized that the study of the above relationships was carried out under static conditions before exercise, during the preparatory period for the competitions. This is because they were looking for an effective and easy-to-measure index for assessing the preparation of the musculoskeletal system for exercise during the starting period in the context of the stabilizing function of the muscles, the task of which is to counteract the overload changes occurring among competitive rowers, widely described in the literature^39–41^.

Various pathological conditions cause changes in blood flow, which can affect skin temperature^25^. Contralateral areas with thermal differences in rest can indicate an increased risk of injury (muscles, joints or tendons)^27^ therefore an analysis of the temperature distribution in terms of its symmetry was also carried out. Some studies point to differences between contralateral body parts as acceptable up to: 0.25°C^42^, 0.5 °C^43^ or 0.62 °C^44^. A larger difference could suggest an inflammatory (hyperthermia) or a degenerative problem (hypothermia)^27^. Marins et al.^27^ defined the difference in temperature ≤ 0.4 °C as “normal”, 0.5–0.7 °C as “monitoring” and 1.1–1.5 °C as “alarm”. A temperature difference greater than 0.8–1 °C may indicate an inflammatory process/injury risk and therefore, according to the above mentioned authors, immediate decrease in training loads or even training suspension and a medical and/or physiotherapeutic assessment of the athlete is necessary.

In our study, in both terms (autumn vs. spring), the difference in temperature measured at rest did not exceed 0.17 °C for the upper and lower limbs, and 0.1 °C for the trunk. As stated above, they were within the range considered "normal". At the same time, it was shown that there was a significant asymmetry of the knee and shoulder extensor muscles at the beginning of the preparatory period, which was no longer significant at the end of this period. In the case of the trunk rotator muscles, a significant difference was observed only in the spring.

It should be noted that any asymmetry^39^ has the effect of increasing susceptibility to injury. However, its reduction in rowers has no clear effect on rowing efficiency, as the biomechanics of rowing says^40,45^ that the athlete during the movement (the phase of pulling the oar) should primarily use the strength of the knee joint extensors and to a lesser extent the back extensor, whose function, in addition to the movement of straightening the trunk, is to the stabilize of the L1-S1 segment of the spine. However, if the extensors are stiffened, at the same time the lumbar region is more loaded and in time overloaded. Taking it into account, it is important to note the positive result, i.e. that the PT value of the trunk extensors increased significantly in the tested group of athletes at the end of the preparatory period. Unfortunately, in the case of the trunk rotators, their asymmetry increased, which is probably due to a significant increase in the torque of the contralateral rotator in spring. At this point, it should be noted that 85% of rowers complain of L1-S1 back pain^41^. Furthermore, the results obtained during an experiment carried out within the framework of our project on an asymmetric rowing ergometer, as presented in Kabaciński et al.^46^, indicate a significant asymmetric electromyography (EMG) activity of the lumbar extensor spine muscle.

Immediately after graded exercise testing in a sweep rowing ergometer, the differences in temperature on both sides of the body were already greater than at rest (in autumn 0.7 °C for the extremities, 0.32 °C for the trunk; in spring 0.22 °C for the extremities, 0.17 °C for the trunk), which were still insignificant, except for one site, i.e. front side of the arms in spring. However, given that the regions of exercised (active) muscles require vasodilation to increase their oxygen supply^11,20^, this may indicate that in the autumn the rowers had more significant asymmetry in the rowing pattern. These results are consistent with the fact that before the measurement point in autumn the athletes were training mainly on the water in a boat (outdoors), which is associated with asymmetric involvement of limb muscles. Whereas in the winter period, they performed workouts on a rowing ergometer and in the gym (indoors), involving muscles more symmetrically. Therefore, one would expect more significant differences between temperatures on the contralateral side area in autumn than in spring. This is in line with Veghte et al.^47^ who showed that during dynamic single-leg ergometry, the skin temperature changes of the exercising leg were more significant than those of the inactive leg, while at the same time, running reduced the skin temperature of both legs. Similarly, Chudecka et al.^36^ showed that in two-oared rowers, for whom training and muscle activity is symmetrical, the surface temperature changes of symmetrical areas were similar after exercise on a rowing ergometer.

It should be noted that the difference in temperatures measured in symmetrical locations in recovery (15 min after exercise) in autumn did not exceed 0.61 °C for the extremities, 0.14 °C for the trunk; in spring 0.57 °C for the extremities, 0.15 °C for the trunk, which tends to support the idea that the recovery processes followed a similar pattern.

Changes in core body and skin temperature during exercise have already been quite well described and examined^10,13,26,32,34^. The initial drop in surface temperature at the beginning of exercise is related to the redistribution of blood to working muscle^10^, while blood flow to non-working tissues is reduced^14^. A moderate increase in body temperature during exercise, especially skeletal muscle temperature, leads to an increase in the rate of chemical reactions, nerve conduction, and conformational changes associated with muscle contractions which can have a beneficial effect on the exercise performance^48^. According to Sargeant et al.^23^ an increase in muscle temperature leads to an increase in maximal power generation (slow twitch fibers take on the characteristics of fast twitch fibers). In contrast, its decrease leads to the opposite effect, i.e., a decrease in muscle power, and may result in injuries.

A decrease in skin temperature immediately after exercise was observed in both terms of examination (autumn and spring), and it was significant compared to the baseline value (before vs. after) except for the front part of the body: contralateral forearm (autumn), ipsilateral forearm (autumn and spring), and ipsilateral arm (spring); and for the back part of the body: contralateral forearm (autumn) and ipsilateral forearm (autumn and spring).

It should be noted that in our study the temperature was measured only 3 times: before, after graded exercise testing and in recovery, so the exact course between these measurement points was not recorded^34^.

Our findings are in line with the results obtained by Merla et al.^11^, who analyzed skin temperature changes during graded exercise in healthy, trained runners. They observed a decrease in whole body temperature at the very beginning of the exercise, the earliest response was in the thighs and forearms. While Fernandes et al.^20^ confirm that depending on the activity of the study area during exercise, there are significant differences in the distribution of skin temperature during exercise.

Blood circulation plays a key role in removing heat from deep areas of the body and transporting heat to the skin^11^. To ensure the dissipation of heat, peripheral vasodilation occurs, and as the blood flow increases^13^, the skin temperature rises. This has been confirmed in conducted studies, immediately after the end of the exercise, an increase in skin temperature to or above the baseline was observed^20,49^. Merla et al.^11^ observed that within a few minutes after graded exercise, the body temperature of runners rose most rapidly in the region of the forearms and fingertips, while other regions (thighs, torso) showed a longer time for the temperature to return to the pre-exercise level.

In recovery (15 min after exercise), the temperature returned to baseline levels (before vs. 15’ after). A significant temperature change was observed in relation to baseline values only at the following locations in the front: contralateral arm (autumn), and contralateral shank (autumn and spring); and in the back: ipsilateral arm (spring). However, for the trunk, 15 min recovery is not enough to reach the baseline temperature, which still remained significantly lower.

These temperature increases observed in the limbs during recovery can be explained by increased blood flow in active muscles in order to improve the heat dissipation to the environment^50^. As suggested, in the recovery phase, there is a decrease in vasoconstrictive activity and increased vasodilatory activity through the action of nitric oxide, acetylcholine, vasoactive intestinal peptide (VIP), and substance P^20^. It must also be taken into account that trained muscle regions have a higher metabolic activity, and the increased blood supply facilitates energy recovery and tissue regeneration. Thus, each body part's temperature response during the recovery period may also be different^21^. So far, the time required for the temperature to return to baseline values after exercise has not been clearly defined. This is related to many internal and external factors, one of which may be the localization in which the measurement was performed. Fernandes et al.^20^ pointed out that significant increases in skin temperature persist for up to 40 min after exercise. They also indicated that each region of interest (ROI) reacts differently to the end of aerobic exercise, and the return of skin temperature to resting values takes relatively long time for some of them.

Our study showed no significant differences in the skin temperatures measured at the beginning (autumn) vs. the end (spring) of the preparatory period. However, a slightly higher resting skin temperature in many locations in spring compared to autumn was observed. At the same time, a tendency towards significantly higher PT values of the hip and knee flexors of both limbs and the trunk rotators were shown. Significant differences were also observed in the extensors of the trunk and flexors of the shoulder joint. In the presence of the finding of significant correlations between the PT of selected muscles and the measurement of resting skin temperature over these muscles, it cannot be excluded that, despite the slight differences in temperature (up to about 0.5 degrees) measured at many sites at the end of the preparatory period, compared to its beginning (autumn vs. spring), higher temperature values, as Bergh and Ekblom^24^ indicated, may have an effect on greater muscle strength.

Studies conducted by other authors have shown that improving aerobic training VO_2_max leads to a lower core temperature threshold for skin vasodilation and sweating, resulting in enhanced heat dissipation^13^, resulting in a lower skin temperature during high-intensity exercise^51^. Measuring changes in aerobic capacity is used to monitor the progress of rowers’ performance during training. On the one hand, as other authors have pointed out, in athletes with a high level of aerobic capacity, the changes under training are small^52^. On the other hand, we cannot exclude that the lack of changes in VO_2_max may be related to using a new sweep rowing ergometer for the graded exercise test (see Supplementary Fig. S1 online), which was designed for this project to evaluate the impact of changes in musculoskeletal overload on athletic performance during the annual training cycle in asymmetric athletes in the context of training optimization.

Our study also found no significant differences in VO_2_max during training, while skin temperature was slightly higher at the end of the preparatory period. This may suggest an adaptive response of the thermoregulatory system during training. Dynamic exercise involving a significant percentage of muscle mass (~ 50%) requires mechanisms related to dissipating more heat, which is partially met by increased cutaneous blood flow^13^. However, this results in an increased demand for cardiac output and additional stress on the circulatory strain. Endurance training leads to the generation of adaptive mechanisms that result in reduced circulatory strain, as well as a shift in the thermoregulatory control of cutaneous blood flow toward higher blood flow levels for a given core body temperature^13^. Moreover, the possible role of local adaptations in the cutaneous microcirculation caused by changes in the concentration of endothelium-derived vasoactive compounds remains unexplored^13^.

It should also be taken into account that the most significant determinant of skin temperature is the ambient temperature^33^. Poland is in a temperate climate zone with considerable temperature differences between November and April. Findings by other authors also suggest that seasonal changes in day length and light intensity may affect some features of temperature regulation^53–55^. Ciuha et al.^53^ noted that when the interval between experimental trials is several months or studies are conducted at different times of the year then the potential effect of seasonal variation on thermoregulatory response must also be considered. Their study observed little change in skin temperature (mean Ts was lower in autumn compared to spring) in young, untrained participants between seasons, suggesting a seasonal adaptation during winter and summer that persisted into spring and autumn.

To summarize, given the high demands placed on professional sports, there is a constant search for new technologies that can be used to monitor training. Research in recent years has shown that infrared thermography is a fast, safe, and highly reproducible technology that can track a subject's temperature in real-time and create a thermal profile^26^. This method can be used to monitor an athlete's thermal response in the context of improving athlete performance and health^26,33^. Due to ongoing research and technological development, this method is constantly progressing, and new research opportunities are being shown, which has also been confirmed in the presented research. However, it is noted that there are influential factors (technical, individual, environmental) that can limit the potential of this method.

Therefore, it is important to consider that our study also has some limitations. One of them is the small study group, which is due to the fact that it was a selected, homogeneous group of highly trained sweep rowers. The authors are recognizing that there are factors affecting temperature measurements that cannot be eliminated such as technical ones related to the accuracy of the device assessing temperature, or individual ones related to the sweat on the surface of the skin at the end of the exercise^55^. In the other hand, some confounding factors can be reduced by standardization, so the Thermographic Imaging in Sports and Exercise Medicine (TISEM) protocol was used for maximum reliability of the study^56^. At the same time, all measurements can present the same measurement error, since every effort was made and the study was performed using the same equipment and methodology, under the same experimental conditions, time of day, and by the same researcher^27^. In addition, differences and temperature changes were measured rather than absolute values, which show individual variability. For this reason, despite its measurement error, thermal imaging is still used as a reliable measurement tool in this type of application^26,34,56^.

In conclusion, thermal imaging allows observing changes in skin temperature and symmetry before and after exercise. At the current stage, it does not appear to be a method that, without supporting other methods such as those assessing muscle function, will allow monitoring of training progress. This requires further research on a larger group of subjects. However, it is expected that in the future, the measurement of skin temperature distribution may become a simple and effective method of assessing the effectiveness of a training program in the context of the stabilizing function of muscle work, instead of the isometric peak torque measurements traditionally used in laboratories to date in highly trained athletes.

## Methods

### Participants

The study involved a total of 14 professional sweep rowers, members of the Polish national rowing team. The study was carried out in two terms of the preparatory period of the rowing training cycle: at the beginning (November) and at the end (March/April). Finally, only 10 males completed the entire study protocol in the two terms and were included in the analysis. All of them were free from pain, injury or inflammation at the time of the experiment.

Each participant was familiarized with all testing procedures and provided a written informed consent prior to the study. Moreover, informed consent from study participants for publication of identifying images in an online open-access publication was obtained. The study protocol was approved by the Ethics Committee for Human Research at the Poznań University of Medical Sciences (approval no. 208/17) and was performed in accordance with the Declaration of Helsinki.

### Training program

The rowers participated in a 24-week training program during the preparatory period (from November to March/April), as described previously^5,6^. The number of training sessions per week ranged from 9 to 10, and from 12 to 14 during training camps.

At the beginning of the preparatory period (microcycles 1 to 4), general training sessions were prevailing with low and moderate intensity aerobic exercises and muscle mass building strength training. The main training modalities in this period were: running, proprioceptive exercises, swimming, cycling, cross-country skiing, team sports, and strength training. The aim of the next mesocycles (microcycles 5 to 12) was developing aerobic endurance, aerobic and anaerobic endurance and maximum strength. During these microcycles rowing on an indoor rower was included. From microcycle 9 aerobic-anaerobic loads (2 sessions per microcycle) increased significantly and power development sessions were introduced. The development of aerobic and aerobic-anaerobic endurance was continued in microcycles 13 to 24 and sessions increasing anaerobic endurance were introduced. From microcycle 17 specific on-water training sessions were included and the amount of such training increased in subsequent microcycles^5,6^.

Research was conducted in the following order:

### Anthropometric measurements

All anthropometric measurements were conducted in the laboratory, in a fasting state, in the morning hours by the same specialist. Body mass and height were measured using a certified digital medical-grade scale and a mechanical measuring rod (WPT 60/150.O, Radwag, Radom, Poland) with the accuracy of 0.1 kg for weight and 0.5 cm for height. Body composition was assessed by the bioimpedance method, using the Tanita BC-418MA Segmental Body Composition Analyzer (Tanita Corporation, Tokyo, Japan).

### Biomechanics measurements

Testing of biomechanical parameters was performed similarly to the thermal examination, in an air-conditioned laboratory, 15 min before the start of the resting skin temperature measurements.

Peak torques of the flexor and extensor muscles of the knee and hip joints were measured in statics on a TBK3-P stand produced by JBA Zb. Staniak (see Supplementary Fig. S2 online). The measuring range of the torque gauge is 1200 Nm, with a maximum measurement error of 1%. The stand's design ensures optimal stabilization of the trunk and knee and hip joints in the sitting position (standard used in biomechanics laboratories). The axis of the rotary torque gauge coincides with the axis of rotation at the joint (Supplementary Fig. S2 and Fig. S3 online). All measurements of peak torques at the above joints were performed using the asymmetric technique, i.e. alternating measurements at the left and right joints. The maximum muscle force during the measurement was released in 1.5–3.0 s. The subject repeated each type of measurement three times with 30 s intervals, while the intervals between measurements at different joints were approximately 30 min. The highest of the 3 recorded peak torques values was used as the final measurement result.

Peak torques of the flexors and extensors of the elbow and shoulder joints were determined in statics using an LR2-P stand from JBA Zb. Staniak (see Supplementary Fig. S3 online). This test bench is equipped with a torque gauge with a measuring range of 500 Nm, with a maximum measurement error of 0.5%. The measurement methodology is analogous to that described above.

Global lower limb extensor strength and peak torques of flexor, extensor and rotator muscles torso were measured in statics, according to the methodology described in Podgórski et al.^6^.

### Thermal examination

The study was performed using the IRT method in accordance with the Thermographic Imaging in Sports and Exercise Medicine (TISEM) checklist to ensure the reliability of thermal imaging research and analysis^57^. The thermographic camera (FLIR Systems Inc., model SC640, Sweden) was used to take pictures. The device has a measuring range of − 40 °C to + 1500 °C, a precision of ± 2 °C or 2% of reading and a resolution of 640 × 380 pixels.

The day before the training session the participants were instructed on how to prepare for the measurement procedure. On the day of the measurements they were not allowed to drink coffee and use other stimulants. The body had to be clean and grease-free; using skin cream or ointment was not allowed. Care was taken to eliminate, in the hours preceding the examination, all external factors that may affect skin temperature, such as physical exertion, exposure to heat or cold.

There was no clothing worn on the scanned body area. The athletes wore running shorts and ankle boots throughout the day of testing. Participants were acclimated for 15 min before the first thermal image. The camera was positioned on a tripod (Manfrotto, Italy) 1 m above the ground and 6 m from the subject. There was a non-reflecting background behind the subject.

**Figure 4:**
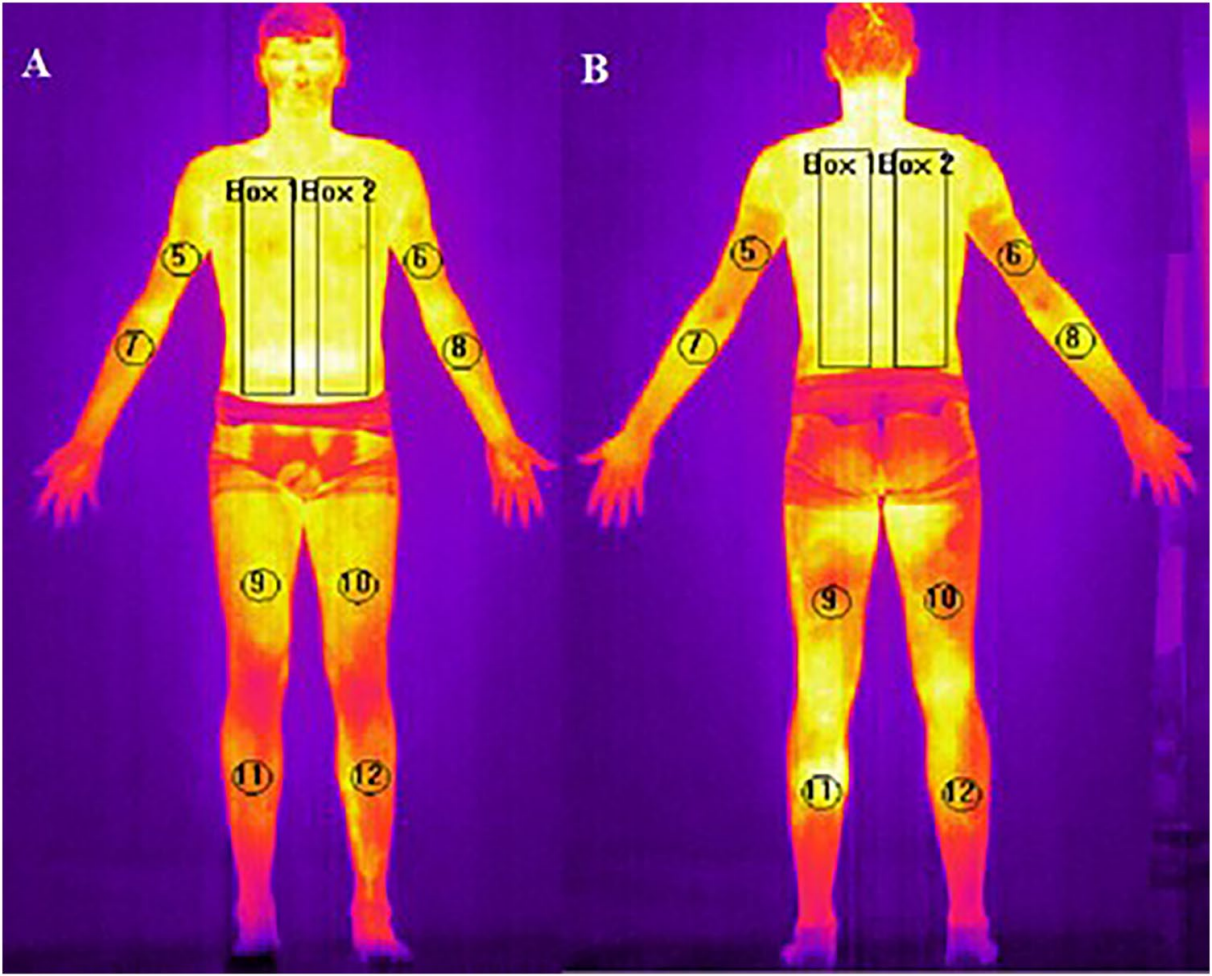
Regions of interest analyzed in each subject. A. Anterior side: Box1/Box2- (right/left chest), 5/6—(right/left arm), 7/8—(right/left forearm), 9/10—(right/left thigh), 11/12—(right/left shinbone); B. Posterior side: Box1/Box2—(right/left back), 5/6—(right/left arm), 7/8—(right/left forearm), 9/10—(right/left thigh), 11/12—(right/left calf).

For the temperature analysis, specified areas on each body part, regions of interest (ROIs), were established. The analyzed body regions were: chest and abdomen, both sides of the back, anterior and posterior right and left arms, and forearms; furthermore, anterior and posterior thighs and legs on the right and left sides. These regions were selected, as shown in Fig. 4. Mean surface temperature of the front and back side of body was calculated using dedicated software (Thermacam Researcher Pro 2.10 software, FLIR, Wilsonville, Oregon, USA).

All procedures were conducted between 8.00 a.m. and noon, in an air-conditioned laboratory (temperature 22 ± 24 °C and humidity 35–44%), 2 h after consuming a light breakfast.

The images of the front and back side of the body were taken three times for each subject: before the session (pre-exercise), immediately after the ergometer test (post-exercise), and after 15 min (recovery). For analysis purpose all participants constituted a single group irrespective of the rowing side and results were recorded as contralateral limb and ipsilateral limb.

All measurements were performed twice by one experienced researcher: I. at the beginning (autumn) and II. at the end (spring) of the preparatory period.

### Graded exercise test protocol

All athletes underwent a graded exercise test (GXT) on a sweep rowing ergometer, as described previously. The ergometer was constructed as part of the research project and was used to simulate the mechanical load on the athlete during the rowing movement (see Supplementary Fig. S1 online). GXT began at a workload of 0.3 W/kg. The workload was incremented by 0.3 W/kg every 3 min until the subject could no longer maintain the workload. Each test lasted 12–18 min depending on the age and aerobic fitness status^5^.

### Statistical analysis

Data were presented as means and standard deviations (SD), medians and interquartile range (Q_1_-Q_3_). The Shapiro–Wilk test was used to check the data for normality of distribution. Assumption on sphericity was tested using Mauchley’s test, verifying if variances of certain variables were identical and equal to respective co-variances. A two-way analysis of variance (ANOVA) for repeated measurements was used to analyze the differences in the skin temperatures (Ts) between autumn and spring. Bonferroni post hoc tests were performed to assess the significance of differences between pairs of measurements. Partial eta-squared (ƞ2) was calculated to determine the effect size. Comparisons of lower limb muscle forces and joint force moments at the two test dates were performed using the Wilcoxon non-parametric test. The same test was used to test the significance of differences in contra- and ipsilateral flexor and extensor force moments. Relationships between joint force moments and temperatures of selected body areas were tested using the non-parametric Spearman rank correlation test. The level of significance was set at < 0.05. Statistical analyses were performed using the Statistica 13.3 software package (TIBCO Software Inc., Palo Alto, CA, USA).

## Supplementary Information

Supplementary Information 1.

Supplementary Information 2.

Supplementary Information 3.

Supplementary Information 4.

Supplementary Information 5.

## Data Availability

The datasets generated during and/or analyzed during the current study are available from the corresponding author on reasonable request.
