# Supplementary material for: The use of thermal imaging for monitoring the training progress of professional male sweep rowers
Source: Sci Rep. 2022 Oct 3;12:16507. doi: 10.1038/s41598-022-20848-7 (PMC9530168; doi:10.1038/s41598-022-20848-7)
Supplement: Supplementary file 1 — Supplementary Information 1. [file 41598_2022_20848_MOESM1_ESM.pdf]

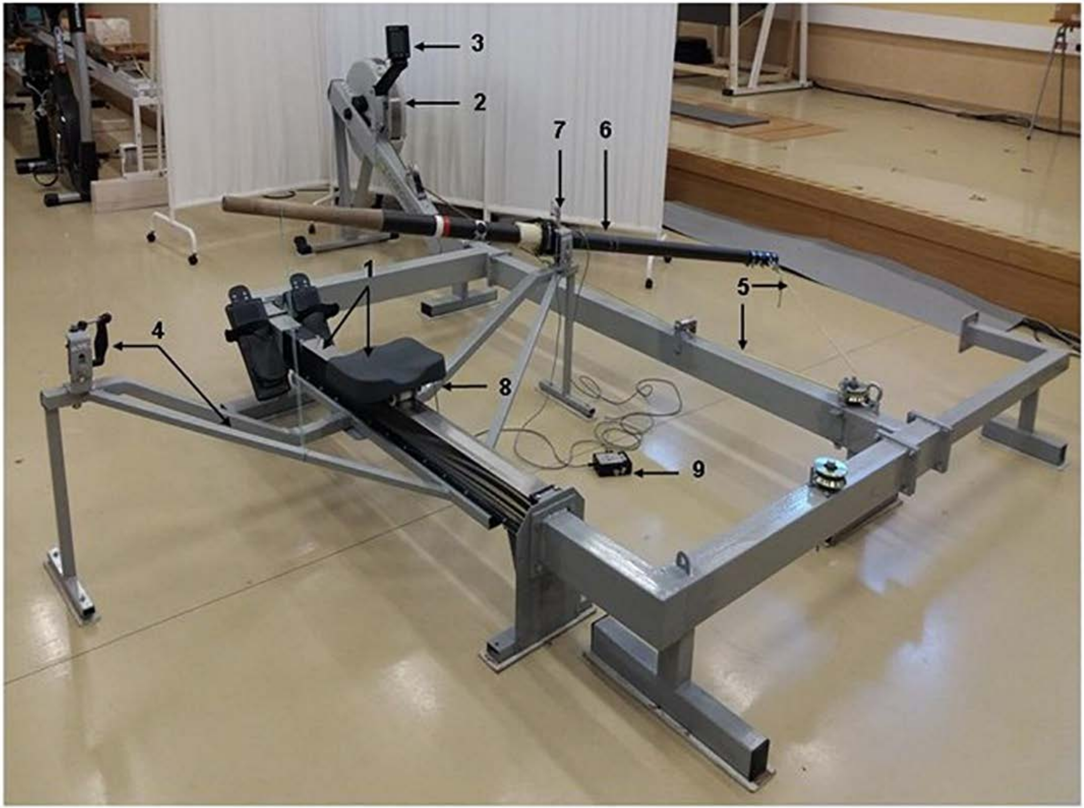

Figure S1. Sweep rowing ergometer

Components of the ergometer: 1. guide rail and seat, 2. oar braking system, 3. LCD monitor displaying oar load parameters, 4. outrigger attached to the guide rail with oarlock, 5. cords providing resistance to the oar, 6. tensometric transducer used to measure the force at the blade, 7. potentiometric transducer at the oarlock measuring the angular position of the oar, 8. accelerometer attached to the seat, 9. recording device for all transducers.
