# Supplementary material for: The use of thermal imaging for monitoring the training progress of professional male sweep rowers
Source: Sci Rep. 2022 Oct 3;12:16507. doi: 10.1038/s41598-022-20848-7 (PMC9530168; doi:10.1038/s41598-022-20848-7)
Supplement: Supplementary file 2 — Supplementary Information 2. [file 41598_2022_20848_MOESM2_ESM.pdf]

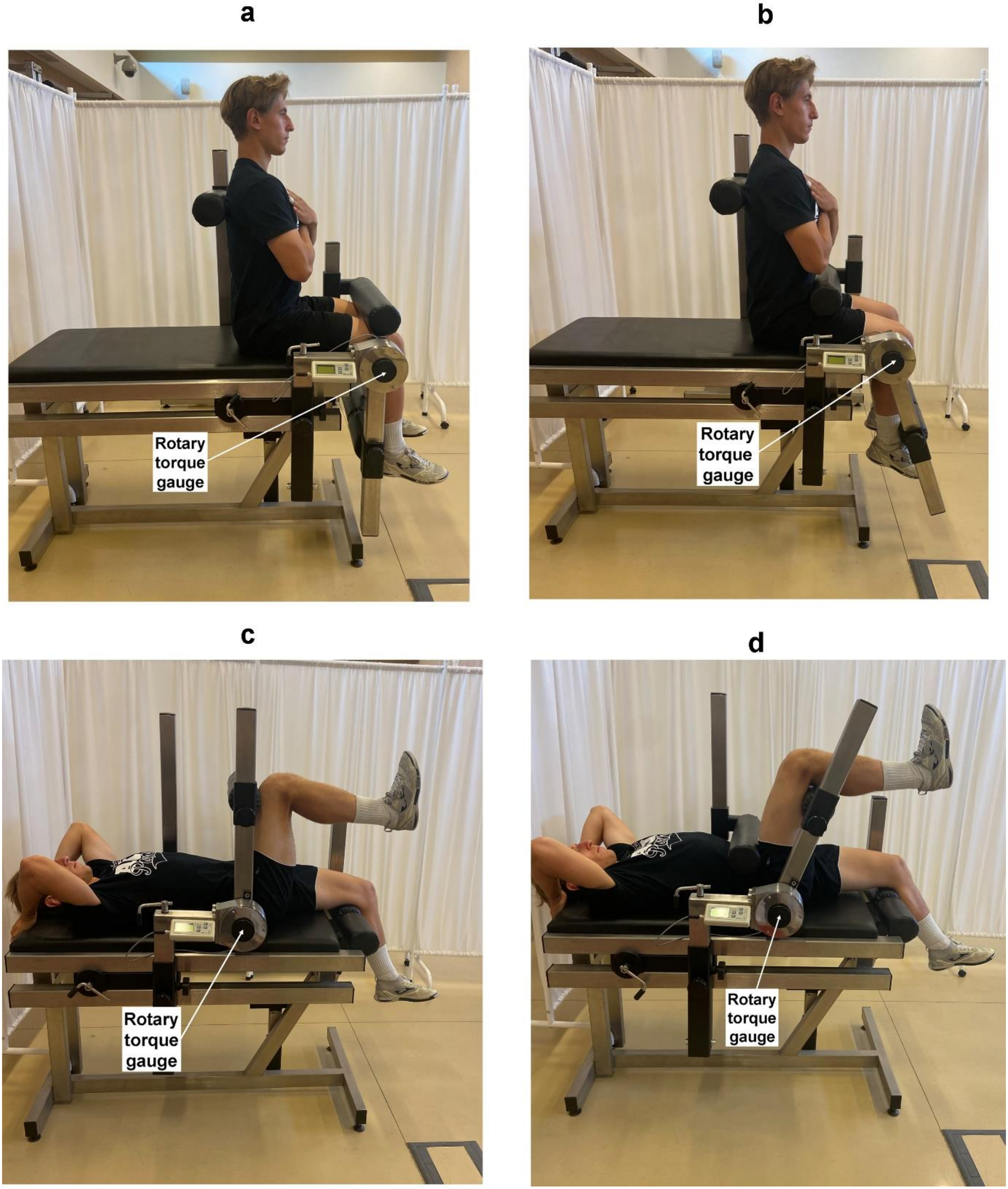

Figure S2. Measurement equipment of the flexors and extensors torque: knee joint (a,b) in the sitting position and hip joint (c,d) in the supine position. The characteristic angles of the torso-thigh and the thigh-shank are 90°
