# Supplementary material for: The use of thermal imaging for monitoring the training progress of professional male sweep rowers
Source: Sci Rep. 2022 Oct 3;12:16507. doi: 10.1038/s41598-022-20848-7 (PMC9530168; doi:10.1038/s41598-022-20848-7)
Supplement: Supplementary file 3 — Supplementary Information 3. [file 41598_2022_20848_MOESM3_ESM.pdf]

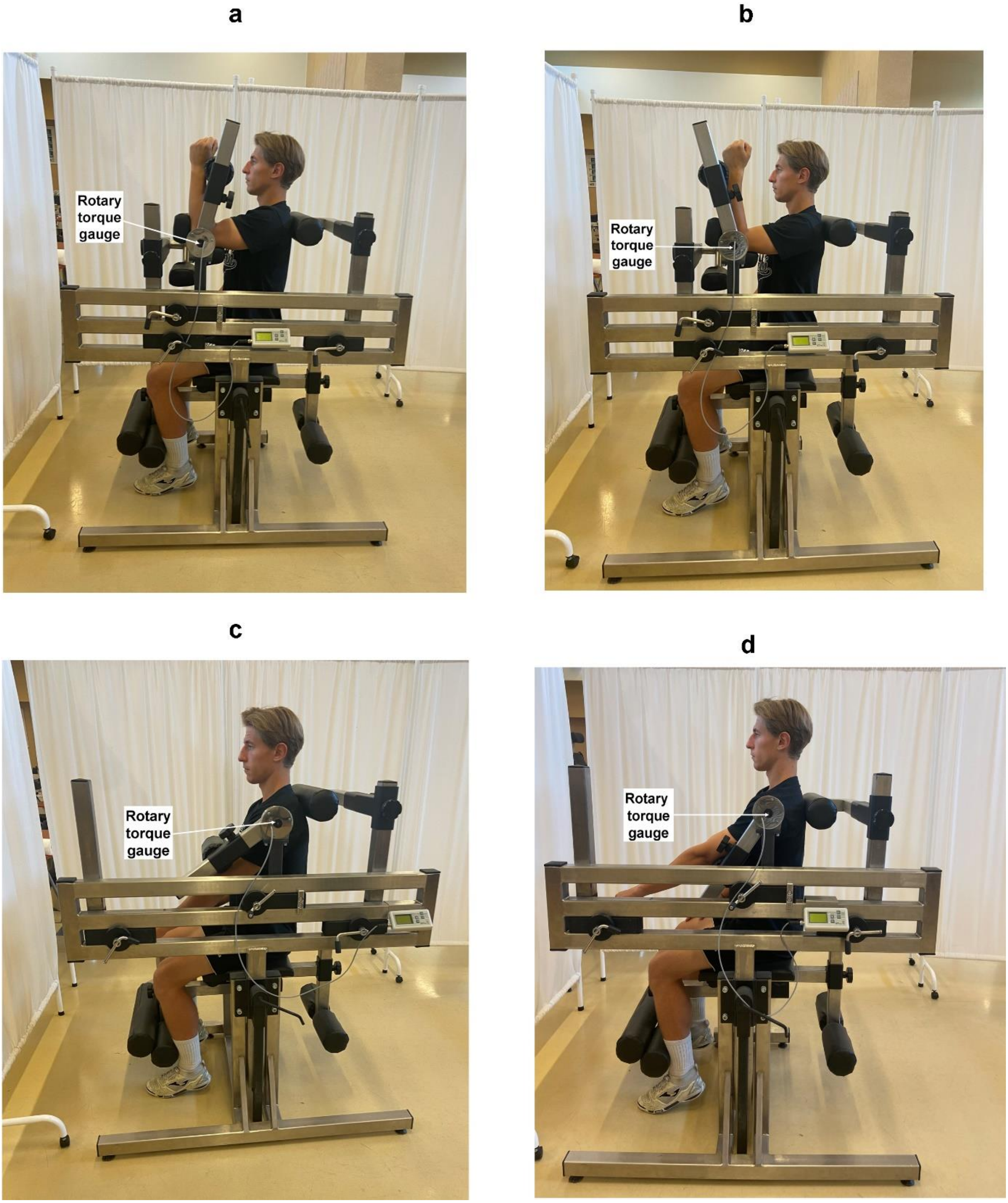

Figure S3. Measurement equipment of the flexors and extensors torque: elbow joint (a,b) and shoulder joint (c,d) in the sitting position. The characteristic angles of the torso-arm and forearm-arm are 90° (a,b), while the torso-arm angle is 45° (c,d)
